# Supplementary material for: RT-LAMP-Based Molecular Diagnostic Set-Up for Rapid Hepatitis C Virus Testing
Source: Biosensors (Basel). 2022 May 5;12(5):298. doi: 10.3390/bios12050298 (PMC9138684; doi:10.3390/bios12050298)
Supplement: Supplementary file 1 [file biosensors-12-00298-s001.zip › biosensors-1685714-supplementary.pdf]

Supplementary Material

RT-LAMP-Based Molecular Diagnostic Set-Up for Rapid Hepatitis C Virus Testing

Sandhya Sharma [1,2], Emmanuel Thomas [3], Massimo Caputi [4] and Waseem Asghar [1,2,5,*]

1 Department of Electrical Engineering and Computer Science, Florida Atlantic University, Boca Raton, FL 33431, USA; ssharma2013@fau.edu
2 Asghar-Lab: Micro and Nanotechnology in Medicine, College of Engineering and Computer Science, Boca Raton, FL 33431, USA
3 Department of Microbiology and Immunology and Sylvester Comprehensive Cancer Center, University of Miami School of Medicine, Miami, FL 33136, USA; ethomas1@med.miami.edu
4 Charles E. Schmidt College of Medicine, Florida Atlantic University, Boca Raton, FL 33431, USA; mcaputi@health.fau.edu
5 Department of Biological Sciences (Courtesy Appointment), Florida Atlantic University, Boca Raton, FL 33431, USA
* Correspondence: wasghar@fau.edu

Table S1. Summary of the existing HCV detection methods illustrating the comparison of target used, detection time and limit of detection (LOD), along with the limitations.

Method	Target	LOD	time	Limitations
Microfluidic chip (from this paper)	RNA	500 copies per mL	45 min	
RT-PCR[1,2]	RNA/DNA	5000 copies per mL	2-4 h	Require expensive thermocycler<br>Time consuming<br>Minute contamination could lead to false negative results
Transcription Mediated Amplification (TMA) [3]	RNA/DNA	1,000 copies/mL	1-2 h	Requires pre-heating<br>Non-specific binding due to low temperature amplification
Rolling Circle Amplification (RCA) [4]	RNA/DNA	1 pmol/L	NA	Non-specific binding of the primers<br>Extensive study is required to validate the RCA method.
Label-free DNA analysis in the microdroplet[5]	DNA	As low as 500 fM	NA	Required excitation source for operation
RNA- oligonucleotide nanoparticle assay[6]	HCV viral protein	1 ng/mL	NA	Centrifugation is required
TaqMan Array Cards (TAC) [7]	RNA	100 IU per/mL	4 h	Low sensitivity in comparison to other assays
Quantum dots- based RNA aptamer system [8]	Viral protein	5 ng/mL	NA	Multiple centrifugation steps are involved
GenMark eSensor HCV genotyping [9]	RNA	175 IU/mL	NA	Contamination issues
Homogeneous electronic monitoring platform[10]	DNA	2.3 pM	NA	It requires immobilization DNA sensing probe and probe labeling
LAMP-based lab-on-disk system [11]	DNA	60 copies per mL	More than 1 h	Complicated equipment is required for operation
Genedrive HCV assay [12]	RNA	2362 IU/mL	NA	Semi-automated system therefore trained personnel are required

Magnetic bead single-stranded DNA glucose-loaded liposomes[13]	RNA	NA	More than 2 h	Require glucose- loaded nanoliposomes
OraQuick HCV Rapid Antibody Test[14,15]	HCV antibodies	20 IU/mL	20-40 min	Lower sensitivity<br>RNA is testing is required to validate the initial results.
Protein microarray and ELISA[16]	HCV antibodies	0.1 ng/mL	20 min	Complex process<br>Sophisticated equipment's are required for processing
Chembio, MedMira, and OraSur [17]	HCV antibodies		Less than 40 min	Weak in-field performance
OTCA and EIA[18]	HCV core antigen	10 000 UI/mL	NA	Low sensitivity and specificity
Magnetic microparticle-based assay[19]	HCV core antigen	10 000 copies/mL	NA	Low sensitivity
Resonant microcantilever arrays[20]	HCV antigen	0.1 ng/mL	30 min	Flow cell must be wet for processing
NAAT [21]	HCV core antigen	500 to 3000 IU/mL	Less than 60 min	Expensive and complex method

Table S2. Microfluidic chip dimensions and thickness of various layers.

	Microfluidic Chip	Thickness layers	Dimensions
**1**	Top- loading layer	750 μm	70 × 75 mm
2	Middle- Well layer	1.5 mm	70 × 75 mm
3	Bottom- Base layer	750 μm	70 × 75 mm
4	Double Sided Adhesive (DSA)	75 μm	70 × 75 mm

Table S3. List of the reagents loaded in the microfluidic chambers along with the volume.

**1**	**Lysis/Binding buffer**	**110 μL**	
**2**	Proteinase K	20 μL	Inlet chamber
**3**	Iso-propanol	30 μL	
**4**	Dyna magnetic beads	15 μL	
**5**	Wash buffer 1 (1:1) with DI water	45 μL	Buffer 1 chamber
**6**	Wash buffer 2 (1:1) with DI water	45 μL	Buffer 2 chamber
**7**	LavaLAMP MasterMix	25 μL	
**8**	HCV RT-LAMP primers	5 μL	
**9**	Elution buffer	19 μL	Reaction chamber
**10**	$MgSO_4$	2.4 μL	
**11**	SYBR green 1 dye	1 μL	
**12**	Mineral oil (14.50 mPa.s at 25 °C)	150 μL (each chamber)	Valving chambers

Table S4. Table illustrating the magnetic actuation time in each chamber.

Chambers	DYNA beads incubation time
Inlet chamber (a)	5 min
Washing buffer 1 (b)	1 min 30 s
Washing buffer 2 (c)	1 min 30 s

Reaction chamber (d)	3 min

Table S5. List of the materials and cost required for the molecular diagnostic set-up fabrication.

	Microfluidic Chip	Cost ($)
1	Poly(methyl methacrylate) (PMMA)	$0.1
2	Double Sided Adhesive (DSA)	$0.1
3	Chip reagent/oil loading	$2
	**TOTAL COST**	**$2.20**
	Assay Runtime	9 min
	**3-D PRINTED PLATFORM ELEMENTS**	
**1**	Arduino Uno R3 Microcontroller (2)	$14.00
**2**	Zip ties	$0.15
**3**	Screws	$0.50
**4**	Aluminum rails	$1.50
**5**	Neodymium Disc Magnets N48	$6.00
**6**	Surface heater	$14.10
**7**	Sensor	$12.99
	**TOTAL COST**	**$49.24**

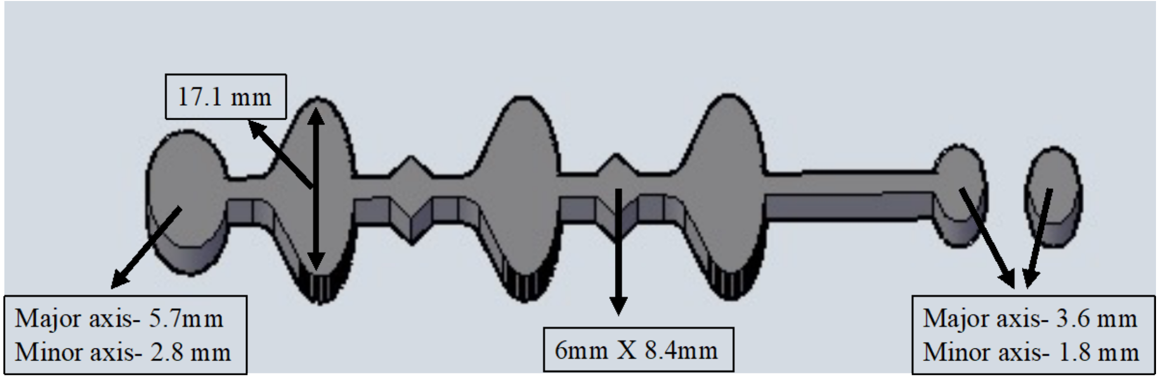

Figure S1. Design of the microfluidic chip illustrating the dimensions of the chip and chambers.

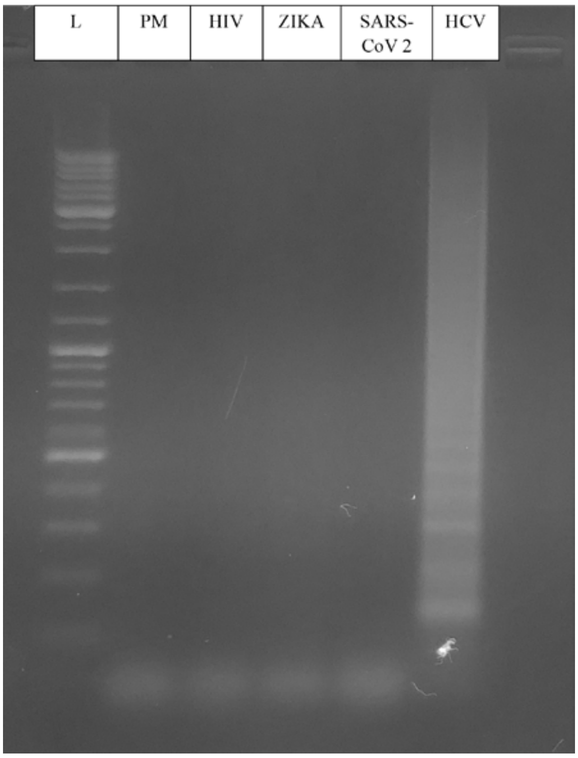

Figure S2. 1.5% gel electrophoresis results stained with Bromophenol blue dye (lane L contains 1 kbp size DNA ladder). Sharp bands in the wells containing the LAMP amplification product of HCV target and no band formation observed in the well holding HIV, ZIKA and SARS-CoV-2 target.

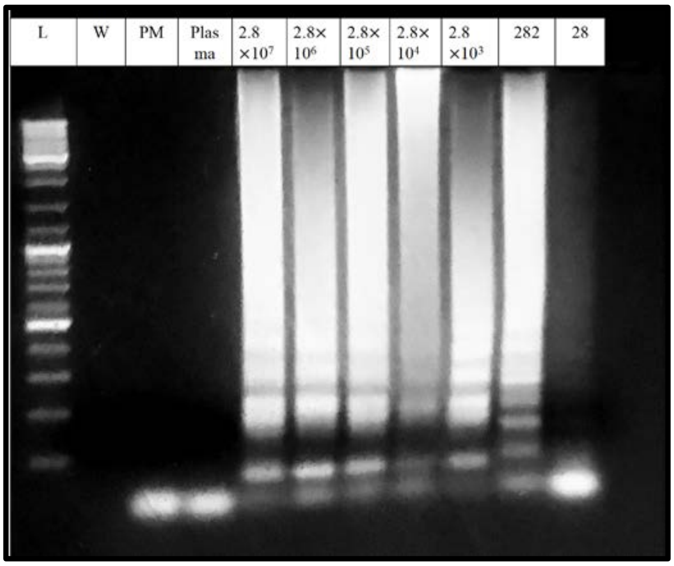

Figure S3. 1.5% gel electrophoresis results stained with Bromophenol blue dye (lane L contains 1 kbp size DNA ladder). Sharp bands in the wells holding the RT-LAMP amplification product of HCV (2.8×10^7 to 282, and slightly with 28 HCV copies/mL) clearly show the specificity of the designed primers and also provide the sensitivity up to 28 HCV copies/mL.

Reference

1. Dietrich, E.A.; Replogle, A.J.; Sheldon, S.W.; Petersen, J.M. Simultaneous Detection and Differentiation of Clinically Relevant Relapsing Fever Borrelia with Semimultiplex Real-Time PCR. *J. Clin. Microbiol.* **2021**, *59*, e0298120.
2. Vázquez-Morón, S.; Ryan, P.; Ardizone-Jiménez, B.; Martín, D.; Troya, J.; Cuevas, G.; Valencia, J.; Jimenez-Sousa, M.A.; Avellón, A.; Resino, S. Evaluation of Dried Blood Spot Samples for Screening of Hepatitis C and Human Immunodeficiency Virus in a Real-World Setting. *Sci. Rep.* **2018**, *8*, 1–6.
3. Sarrazin, C.; Teuber, G.; Kokka, R.; Rabenau, H.; Zeuzem, S. Detection of Residual Hepatitis C Virus RNA by Transcription-Mediated Amplification in Patients with Complete Virologic Response According to Polymerase Chain Reaction–Based Assays. *Hepatology* **2000**, *32*, 818–823.

4. Ji, M.H.; Hu, G.F.; Zheng, Y.; Gu, D.Y.; Long, J.; Lu, W.P.; He, J.A.; Tan, S.Q.; Shi, L.; Liu, C.X.; et al. Surface Plasmon Resonance Technology Combined with Rolling Circle Amplification for Detection of Hepatitis C Virus. *J. Shanghai Jiaotong Univ. (Med. Sci.)* **2012**, *32*, 693.
5. Narayanamurthy, V.; Jeroish, Z.E.; Bhuvaneshwari, K.S.; Samsuri, F. Hepatitis C Virus (HCV) Diagnosis: Via Microfluidics. *Anal. Methods* **2021**, *13*, 740–763.
6. Firdaus, R.; Saha, K.; Biswas, A.; Sadhukhan, P.C. Current Molecular Methods for the Detection of Hepatitis C Virus in High Risk Group Population: A Systematic Review. *World J. Virol.* **2015**, *4*, 25.
7. Pauly, M.D.; Kamili, S.; Hayden, T.M. Impact of Nucleic Acid Extraction Platforms on Hepatitis Virus Genome Detection. *J. Virol. Methods* **2019**, *273*, 113715.
8. Warkad, S.D.; Song, K.S.; Pal, D.; Nimse, S.B. Developments in the HCV Screening Technologies Based on the Detection of Antigens and Antibodies. *Sensors* **2019**, *19*, 4257. https://doi.org/10.3390/S19194257.
9. Sam, S.S.; Steinmetz, H.B.; Tsongalis, G.J.; Tafe, L.J.; Lefferts, J.A. Validation of a Solid-Phase Electrochemical Array for Genotyping Hepatitis C Virus. *Exp. Mol. Pathol.* **2013**, *95*, 18–22.
10. Lu, M.; Xu, L.; Zhang, X.; Xiao, R.; Wang, Y. Ag(I)-Coordinated Hairpin DNA for Homogenous Electronic Monitoring of Hepatitis C Virus Accompanying Isothermal Cycling Signal Amplification Strategy. *Biosens. Bioelectron.* **2015**, *73*, 195–201.
11. Zhuang, J.; Yin, J.; Lv, S.; Wang, B.; Mu, Y. Advanced "Lab-on-a-Chip" to Detect Viruses—Current Challenges and Future Perspectives. *Biosens. Bioelectron.* **2020**, *163*, 112291.
12. Llibre, A.; Shimakawa, Y.; Mottez, E.; Ainsworth, S.; Buivan, T.P.; Firth, R.; Harrison, E.; Rosenberg, A.R.; Meritet, J.F.; Fontanet, A.; et al. Development and Clinical Validation of the Genedrive Point-of-Care Test for Qualitative Detection of Hepatitis C Virus. *Gut* **2018**, *67*, 2017–2024.
13. Monteail, S.; Casson, A.J.; Jones, S.T. Electronic and electrochemical viral detection for point-of-care use: A systematic review. *PLoS ONE* **2021**, *16*, e0258002. https://doi.org/10.1371/journal.pone.0258002.
14. Lee, S.R.; Kardos, K.W.; Schiff, E.; Berne, C.A.; Mounzer, K.; Banks, A.T.; Tatum, H.A.; Friel, T.J.; Demicco, M.P.; Lee, W.M.; et al. Protocols Evaluation of a New, Rapid Test for Detecting HCV Infection, Suitable for Use with Blood or Oral Fluid. *J. Virol. Methods* **2010**, *172*, 27–31.
15. Gao, F.; Talbot, E.A.; Loring, C.H.; Power, J.J.; Dionne-Odom, J.; Alroy-Preis, S.; Jackson, P.; Bean, C.L. Performance of the OraQuick HCV Rapid Antibody Test for Screening Exposed Patients in a Hepatitis C Outbreak Investigation. *J. Clin. Microbiol.* **2014**, *52*, 2650.
16. Xu, R.; Gan, X.; Fang, Y.; Zheng, S.; Dong, Q. A Simple, Rapid, and Sensitive Integrated Protein Microarray for Simultaneous Detection of Multiple Antigens and Antibodies of Five Human Hepatitis Viruses (HBV, HCV, HDV, HEV, and HGV). *Anal. Biochem.* **2007**, *362*, 69–75.
17. Smith, B.D.; Teshale, E.; Jewett, A.; Weinbaum, C.M.; Neaigus, A.; Hagan, H.; Jenness, S.M.; Melville, S.K.; Burt, R.; Thiede, H.; et al. Performance of Premarket Rapid Hepatitis C Virus Antibody Assays in 4 National Human Immunodeficiency Virus Behavioral Surveillance System Sites. *Clin. Infect. Dis.* **2011**, *53*, 780–786.
18. Yu, Y.C.; Wang, Y.; He, C.L.; Wang, M.R.; Wang, Y.M. Management of Hepatitis C Virus Infection in Hemodialysis Patients. *World J. Hepatol.* **2014**, *6*, 419.
19. Leary, T.P.; Gutierrez, R.A.; Muerhoff, A.S.; Birkenmeyer, L.G.; Desai, S.M.; Dawson, G.J. A Chemiluminescent, Magnetic Particle-Based Immunoassay for the Detection of Hepatitis C Virus Core Antigen in Human Serum or Plasma. *J. Med. Virol.* **2006**, *78*, 1436–1440.
20. Timurdogan, E.; Alaca, B.E.; Kavakli, I.H.; Urey, H. MEMS Biosensor for Detection of Hepatitis A and C Viruses in Serum. *Biosens. Bioelectron.* **2011**, *28*, 189–194.
21. Fourati, S.; Feld, J.J.; Chevaliez, S.; Luhmann, N. Approaches for Simplified HCV Diagnostic Algorithms. *J. Int. AIDS Soc.* **2018**, *21*, e25058.

.
